# Hypothalamic-pituitary-gonadal axis disturbance and its association with insulin resistance in kidney transplant recipients

**DOI:** 10.1590/2175-8239-JBN-2021-0250en

**Published:** 2022-05-23

**Authors:** Lourdes Balcázar-Hernández, Victoria Mendoza-Zubieta, Baldomero González-Virla, Brenda González-García, Mariana Osorio-Olvera, Jesús Ubaldo Peñaloza-Juarez, Irene Irisson-Mora, Martha Cruz-López, Raúl Rodríguez-Gómez, Ramón Espinoza-Pérez, Guadalupe Vargas-Ortega

**Affiliations:** 1 Instituto Mexicano del Seguro Social, Centro Médico Nacional Siglo XXI, Hospital de Especialidades, Endocrinology Department, México City, Mexico.; 2 Universidad Nacional Autónoma de México, Facultad de Medicina, Mexico City, Mexico.; 3 Instituto Mexicano del Seguro Social, Centro Médico Nacional Siglo XXI, Hospital de Especialidades, Kidney Transplant Unit, México City, Mexico.

**Keywords:** Kidney Transplantation, Hy- pogonadism, Estradiol, Testosterone, Go- nadotropins, Hyperinsulinism, Transplante Renal, hipogonadismo, estradiol, testosterona, gonadotrofinas, hiperinsulinemia

## Abstract

**Objective:**

To evaluate hypothalamic-pi- tuitary-gonadal (HPG) axis alterations at 1 and 12 months after kidney transplan- tation (KT) and their association with in- sulin resistance.

**Methods:**

A retrospective clinical study was conducted in a tertiary care center in kidney transplantation recipients (KTRs) aged 18- 50 years with primary kidney disease and stable renal graft function. LH, FSH, E2/T, and HOMA-IR were assessed at 1 and 12 months after KT.

**Results:**

Twenty-five KTRs were included; 53% were men, and the mean age was 30.6±7.7 years. BMI was 22.3 (20.4-24.6) kg/m2, and 36% had hypogonadism at 1 month vs 8% at 12 months (p=0.001). Re- mission of hypogonadism was observed in all men, while in women, hypogonadotropic hypogonadism persisted in two KTRs at 12 months. A positive correlation between go- nadotrophins and age at 1 and 12 months was evident. Fifty-six percent of patients had insulin resistance (IR) at 1 month and 36% at 12 months (p=0.256). HOMA-IR showed a negative correlation with E2 (r=- 0.60; p=0.050) and T (r=-0.709; p=0.049) at 1 month, with no correlation at 12 months. HOMA-IR at 12 months after KT correlated positively with BMI (r=0.52; p=0.011) and tacrolimus dose (r=0.53; p=0.016).

**Conclusion:**

Successful KT restores the HPG axis in the first year. Hypogonadism had a negative correlation with IR in the early pe- riod after KT, but it was not significant at 12 months.

## Resumo

**Objetivo:**

Avaliar as alterações do eixo hipotálamo-hipófise-gonadal (HHG) em 1 e 12 meses após transplante renal (TR) e sua associação com a resistência à insulina.

**Métodos:**

Foi realizado um estudo clínico retrospectivo em um centro de cuidados terciários em receptores de transplante renal (RTR) com idade entre 18-50 anos com doença renal primária e função do enxerto renal estável. LH, FSH, E2/T e HOMA-IR foram avaliados em 1 e 12 meses após o TR.

**Resultados:**

foram incluídos 25 RTR; 53% eram homens e a média de idade foi de 30,6±7,7 anos. O IMC foi de 22,3 (20,4-24,6) kg/m^2^ e 36% apresentaram hipogonadismo em 1 mês *vs* 8% aos 12 meses (p=0,001). A remissão do hipogonadismo foi observada em todos os homens, enquanto nas mulheres, o hipogonadismo hipogonadotrófico persistiu em dois RTR aos 12 meses. Ficou evidente uma correlação positiva entre gonadotrofinas e idade em 1 e 12 meses. Cinquenta e seis por cento dos pacientes apresentaram resistência à insulina (RI) em 1 mês e 36% aos 12 meses (p=0,256). O HOMA-IR mostrou uma correlação negativa com E2 (r=-0,60; p=0,050) e T (r=-0,709; p=0,049) em 1 mês, sem correlação em 12 meses. O HOMA-IR aos 12 meses após TR correlacionou-se positivamente com o IMC (r=0,52; p=0,011) e a dose de tacrolimus (r=0,53; p=0,016).

**Conclusão:**

O TR bem-sucedido restaura o eixo HHG no primeiro ano. O hipogonadismo apresentou uma correlação negativa com a RI no período inicial após o TR, mas essa correlação não foi significativa aos 12 meses.

## Introduction

Hypothalamic-pituitary-gonadal (HPG) axis disorders, such as infertility, sexual dysfunction, impotence, libido loss, anovulation, and impaired spermatogenesis, are common in women and men with chronic kidney disease (CKD). The pathogenesis of HPG axis disorders in CKD includes inhibition of LH signaling in the uremic state, direct toxic effects of uremia on the gonads, presence of hyperprolactinemia (due to increased production and reduced renal clearance of prolactin), and alterations in gonadal feedback. Obesity, diabetes, and drugs such as glucocorticoids may also contribute. Hypogonadism has been linked to bone disease, increased cardiovascular risk, insulin resistance, infertility, immune system disorders, and reduced quality of life^1,2^.

Successful kidney transplantation (KT) has been associated with the restoration of HPG axis function and fertility; however, some kidney transplantation recipients (KTRs) may persist with hypogonadism^1,2^. Hypogonadism is associated with bone disease, high cardiovascular risk and metabolic disease in the nonkidney transplant population; these bone and cardio-metabolic diseases may be present in KTRs, worsening the prognosis. Information about hypogonadism and its association with metabolic disease is scarce in KTRs. The aim of this study was to evaluate HPG axis alterations at 1 and 12 months after KT and their association with insulin resistance.

## Materials and Methods

### Patients

A retrospective clinical study was conducted in a cohort of KTRs. Eligible patients were men and women aged 18-50 years with a history of CKD due to primary kidney disease, stable renal graft function, and an estimated glomerular filtration rate (eGFR) >60 mL/min at 12 months after KT who were enrolled in the Bone Mineral Metabolism Clinic of the Hospital de Especialidades, Centro Médico Nacional Siglo XXI in Mexico City. Patients with diabetes, autoimmune diseases, overweight, obesity, neoplasms, graft dysfunction or rejection, postmenopausal women, women taking contraceptives, and men and women receiving hormone replacement therapy due to a diagnosis of hypogonadism before the start of the study were excluded. All patients were receiving immunosuppressive treatment with corticosteroids (prednisone) associated with mycophenolate mofetil and tacrolimus.

Amenorrhea was defined as the cessation of regular menses for three months. The presence of sexual dysfunction was established according to the Diagnostic and Statistical Manual of Mental Disorders (5th edition)^3^; eGFR was assessed using the CKD-EPI equation.

Assessment of the HPG axis included determination of luteinizing hormone (LH), follicle stimulating hormone (FSH), estradiol (E2), and testosterone (T) at 1 and 12 months after KT. Hypergonadotropic hypogonadism was defined as E2 levels <20 pg/ mL in women and T levels <300 ng/dL in men, in addition to elevated LH and FSH. Insulin sensitivity was assessed at 1 month and 12 months after KT using the homeostasis model for insulin resistance (HOMA-IR) and was calculated as follows: [fasting insulin × fasting glucose (mg/dL)/405]. Subjects were considered insulin resistant when the HOMA-IR score was > 2.5^4^.

### Biochemical measurements

For biochemical determinations, 6 mL of blood was collected in BD Vacutainer tubes (BD Franklin Lakes, New Jersey, USA) and centrifuged at 3150 × g for 15 min in an Allegra X-22 centrifuge (Beckman Coulter Inc, USA) to obtain serum, which was analyzed with a COBAS glucose and insulin measurement kit (2010 Roche Diagnostics, Indianapolis, USA) using a photocolorimetry technique and a Roche Modular P800 spectrophotometer (2010 Roche Diagnostics, Indianapolis, USA). LH, FSH, prolactin, E2, and T were measured using the radioimmunoassay technique, COBAS (Roche Diagnostics, Indianapolis, USA). Normal reference ranges were FSH 3.5-12.5 mIU/mL; LH 2.4-12.6 mIU/mL; prolactin 5-35 ng/ mL; glucose 65-110 mg/dL; and insulin 3.21-16.32 mIU/mL.

### Statistical Analysis

Continuous variables are described as the mean ± standard deviation (SD) or median and interquartile range (IQR) according to their distribution. For categorical variables, proportions (expected frequency, prevalence) were used. Student’s t test, Mann-Whitney U test, or Wilcoxon signed-rank test were used to establish the association between continuous variables, and the χ2 test was used for categorical variables. Correlations of quantitative variables were performed using Spearman’s rank test or Pearson’s product-moment test.

To establish a statistically significant association, a two-sided p value < 0.05 was considered. The statistical packages SPSS Statistics V25.0 (IBM SPSS^®^, USA) and STATA V14 (StataCorp^®^, USA) were used.

## Results

### Characteristics of the Kidney Transplant Recipients

A total of 25 KTRs were included, of which 53% (n=13) were men, and the mean age was 30.6±7.7 years. BMI was 22.3 (20.4-24.6) kg/m^2^. Congenital abnormalities of the kidney and urinary tract were the most common etiologies of CKD. Most patients had a related living donor kidney transplant. The eGFR was 81.85 (67.2-110) mL/min/SC by CKD- EPI at 1 month versus 77.30 (60.2-94.6) mL/min/ SC at 12 months after KT (p=0.108). All patients received mycophenolate, prednisone, and tacrolimus as immunosuppressive therapy. The baseline characteristics of the KTRs included in the study are summarized in Table 1.

**Table 1:** Baseline characteristics of kidney transplant recipients included in the study

Baseline characteristics of kidney transplant recipients	
Sex; %(n=)	Women: 48 (12)
	Men: 52 (13)
Age (years); mean±SD	30.6±7.7
Ethnicity; (n=)	Latins (25)
BMI (kg/m^2^); M (IQR)	22.3 (20.4-24.6)
Tobacco consumption; % (n=)	Pretransplantation: 20 (5) Post-transplantation: 0
Alcohol consumption; %(n=)	Pretransplantation: 24 (6)
	Post-transplantation: 0
Chronic Kidney Disease Etiology; %(n=)	CAKUT: 44 (11) Uncertain: 36 (9)
	Glomerulonephritis (membranous, proliferative and focal segmental glomerulosclerosis): 12 (3)
	Others: 8 (2)
KDIGO Classification Stage before kidney; %(n=) transplantation	Stage 4: 4 (1)
	Stage 5: 96 (24)
Type of dialysis before kidney transplantation; %(n=)	Peritoneal Dialysis: 50 (12) Hemodialysis: 12.5 (3)
	Hemodialysis after peritoneal dialysis: 37.5 (9)
Dialysis duration (months); M (IQR)	39 (12-69.6)
Type of Kidney Transplant; % (n=)	Related living-donor kidney transplant: 44 (11) Unrelated Living-donor kidney transplant: 24 (6)
	Deceased donor kidney transplant: 32 (8)
Immunosuppressive treatment dose 12 months after kidney transplantation (mg/d); M(IQR)	Mycophenolate: 1500 (1000-2000)
	Prednisone: 15 (10-15)
	Tacrolimus: 6.0 (5-8)

CAKUT: congenital abnormalities of the kidney and urinary tract

X: median, SD: standard deviation, M: median; IQR: interquartile range.

### Hypothalamic-Pituitary-Gonadal Axis

Amenorrhea and female sexual interest/arousal disorder occurred in 33% (n=4) and 17% (n=2) of women at 1 and 12 months after TK, respectively. The most common sexual dysfunction in men was erectile dysfunction, with a frequency of 38% (n=5) at 1 month after KT. There was no evidence of male sexual dysfunction at 12 months after KT.

There were significant differences in gonadal steroids at 1 month vs 12 months in the total sample [T in men: 354 (266-503) vs 457 (394-568) ng/dL; p=0.003 and E2 in women: 30.7 (5-67) vs 60.4 (42-203) pg/mL; p=0.041]. Hypogonadism was present in 36% of KTRs in the first month; 28% had hypogonadotropic hypogonadism, and two KTRs had hypergonadotropic hypogonadism.

A decrease in the frequency of hypogonadism was evident 12 months after KT (36% vs 8%; p= 0.001). There was no change in gonadotrophins or prolactin (Table 2).

**Table 2:** Characteristics of hypothalamic-pituitary-gonadal axis and homa-ir in kidney transplant recipients

	At 1 month after KT	At 12 months after KT	p
LH (mcIU/mL); M (IQR)	6.8 (4.7-13.1)	7.4 (5.3-17.8)	0.304
FSH (mcIU/mL); M (IQR)	4.6 (3.4-11.5)	5.5 (3.7-9.05)	0.391
Gonadal Steroids; M(IQR)			
Estradiol (in women) (pg/mL)	60.2 (5-67)	60.4 (42-203)	0.041*
Testosterone (in men) (ng/dL)	354 (266-503)	457 (394-568)	0.003*
Prolactin (ng/mL); M(IQR)	18.5 (14.3-22.8)	17.7 (15.9-23)	0.968
Hypogonadism; %(n)	36 (9)	8 (2)	0.001*
Type of hypogonadism; %(n)			
Hypergonadotropic hypogonadism	8 (2)	8 (2)	
Hypogonadotropic hypogonadism	28 (7)	0	
HOMA-IR; M(IQR)	2.64 (1.76-2.99)	2.30 (1.54-2.83)	0.546
Insulin resistance; %(n)	56 (14)	36 (9)	0.162

* p<0.05; SD: standard deviation, M: median; IQR: interquartile range; KT: kidney transplantation.

A decrease in the frequency of hypogonadism at 12 months of KT was present in both women (33% vs 17%) and men (38% vs 0%). Hypogonadism remission was observed in all men. Two women with hypergonadotropic hypogonadism persisted with this alteration of the HPG axis at 1 year after KT (Table 3). The two patients with hypogonadism received combined estrogen and progesterone hormone replacement therapy.

**Table 3:** Gonadotropic axis and homa-ir characteristics according to sex at 1 and 12 months after kidney Transplantation

	Women (n=12)		Men (n=13)	
	At 1 month after KT	At 12 months after KT	At 1 month after KT	At 12 months after KT
LH (mcIU/mL); M (IQR)	7.8 (4.7-18.6)	12.5 (5.9-20.3)	6.8 (4.7-10.8)	5.5 (4.3-12.5)
FSH (mcIU/mL); M (IQR)	4.4 (2.8-8.7)	5.5 (3.5-8.7)	4.6 (4.1-12.8)	5.6 (3.9-9.6)
Gonadal Steroids; M (IQR)				
Estradiol (in women) (pg/mL)	60.2 (5-67)	60.4 (42-203)*	354 (266-503)	457 (394-568) *
Testosterone (in men) (ng/dL)				
Prolactin (ng/mL); M (IQR)	17.9 (14.2-24.6)	19.3 (15.6-23)	18.6 (14.3-22.3)	17.7 (16.3-21.9)
Hypogonadism; % (n)	33 (4)	17 (2) *	38 (5)	0 *
Type of hypogonadism; % (n)				
Hypogonadotropic hypogonadism	17 (2)	17 (2)	0	0
Hypergonadotropic hypogonadism	25 (3)	0	38.5 (5)	0
HOMA-IR; M (IQR)	1.97 (1.38-2.65)	2.02 (1.46-2.73)	2.80 (2.31-3.59	2.3 (1.74-3.01)
Insulin resistance; % (n)	42 (5)	33(4)	69(9)	38(5)

* p<0.05; SD: standard deviation, M: median; IQR: interquartile range; KT: kidney transplantation.

At 1 month after KT, positive correlations between LH and FSH [r=0.98; p=0.001) and age and LH [r=0.54; p=0.008) and FSH [r=0.55; p=0.007) were found. At 12 months after KT, positive correlations were evident between LH and FSH (r=0.96; p=0.001) and age and LH (r=0.96; p=0.001) and FSH (r=0.58; p=0.018).

FSH at 12 months showed a positive correlation with FSH [r=0.99; p=0.001) and LH [r=0.99; p=0.001) at 1 month. LH at 12 months was positively correlated with FSH [r=0.97; p=0.001) and LH at 1 month (r=0.96; p=0.001); prolactin at 12 months was positively correlated with prolactin at 1 month (r=0.50; p=0.010). Gonadal steroids showed no correlation with gonadotrophins or doses of immunosuppressive agents. The presence of hypogonadism was not related to eGFR or doses of immunosuppressive agents.

### Homa-IR, Insulin Resistance, and HPG Axis

In the total sample, HOMA-IR at 1 month was 2.64 (1.76-2.99) vs 2.30 (1.54-2.83) at 12 months (p=0.546); insulin resistance (IR) was evident in 56% (n=14) of patients at 1 month and 36% (n=9) at 12 months (p=0.256) (Table 2). In women, IR was evident in 42% at 1 month versus 33% at 12 months (p=0.99); in men, IR was observed in 69% at 1 month versus 38% at 12 months (p=0.237) (Table 3). At 1 month, HOMA-IR in women was 1.97 (1.38-2.65) versus 2.80 (2.31-3.59) in men (p=0.376). At 12 months, the HOMA-IR in women was 2.02 (IQR 1.46-2.73) versus 2.3 (IQR 1.74-3.01) in men (p=0.026). At 1 month after KT, HOMA-IR showed a negative correlation with E2 (r=-0.60, p=0.050) and T (r=-0.709, p=0.049). No correlation was found between HOMA-IR and E2 (p=0.352) or T (p=0.150) at 12 months.

HOMA-IR at 1 month after TK had no correlation with BMI (r=0.25; p=0.09), dialysis duration (r=0.11; p=0.55), creatinine (r=0.11; p=0.55), or eGFR (r=0.11; p=0.46). HOMA-IR at 12 months post-KT was positively correlated with BMI (r=0.52; p=0.011) and tacrolimus dose (r=0.53; p=0.016). There was no association with creatinine (r=0.11; p=0.59), eGFR (r=0.17; p=0.41), dialysis duration (r=0.27; p=0.28), doses of prednisone (r=0.16; p=0.44) or mycophenolate (r=0.38; p=0.07).

## Discussion

Disturbances in the HPG axis are common in CKD. The specific effects of CKD on the HPG axis in women include impaired ovulation (anovulation, cycle disruptions, hypoestrogenism, and low progesterone levels), dysfunctional uterine bleeding, hyperprolactinemia (increased production and reduced clearance), and menopause. In men, disturbances include impaired spermatogenesis (reduction of ejaculate volume and percentage of motile sperm, oligospermia or azospermia),testiculardamage(reducednumberofmature spermatocytes, aplasia of germinal elements, atrophy of Sertoli cells, interstitial fibrosis and calcifications), impaired gonadal steroidogenesis, disruption of gonadotropin release, and hyperprolactinemia.

Successful KT can restore HPG axis function^1,2^. Three to 12 months after KT, levels of FSH, LH and plasma testosterone, androgens (in men) or estradiol (in women) are restored toward the normal range^5^. Usually, at 3-4 months after KT, there is improvement in sexual function and fertility^6^. In men, it was found that sperm morphology and density did not change after KT; however, sperm motility improved. Normalization of LH, FSH, and T and improvement of sexual function were evident^7^. Reinhardt et al. reported rapid recovery of male hypogonadism within 3 months after KT, predominantly in young patients. In their study, 18% of KTRs had hypogonadism at 1 year after KT, mostly in older patients; a decrease in the E2/T ratio and normalization of prolactin were evident 4 weeks after KT^8^.

In our series, we found an improvement in hypogonadism in the first year after KT. The positive relationship between LH, FSH, and age allows us to propose that higher gonadotropin levels at 1 month after KT and older age would predict higher LH and FSH levels at 1 year. Persistence of hypogonadism after 1 year of KT was evidenced in 8%, lower than the reported frequency, highlighting that KTRs with hypergonadotropic hypogonadism at 1 month of KT were the only ones who persisted with HPG axis alterations.

Several factors, such as immunosuppression or comorbidities, have been associated with a lack of improvement in HPG axis function and persistent hypogonadism. In our study, there was no evidence of a relationship between hypogonadism, renal function, prolactin levels, or doses of immunosuppressive agents.

Hypogonadism has been associated with bone disease, increased cardiovascular risk, insulin resistance, infertility, immune system disorders and decreased quality of life^9^. Some of these alterations have been found in KTRs, such as decreased virility, insulin resistance, weight gain and new-onset diabetes. Older age, family history of diabetes, ethnicity, obesity, sedentary lifestyle, viral infections, and immunosuppressive treatment are associated risk factors.^10,11^

The detection of IR and its related factors is essential, especially in KTRs. IR is associated with hyperinsulinemia, hyperglycemia, elevated adipokine levels, vascular endothelial dysfunction, abnormal lipid profiles, hypertension, and vascular inflammation, all of which favor the development of atherosclerotic cardiovascular disease^12^. In our series, more than half of the patients had IR, predominantly men, with a high frequency persisting at 12 months after KT despite dietary modifications during follow- up. We highlight the positive association of HOMA- IR with BMI and tacrolimus dose at 12 months after KT. Calcineurin inhibitors, including tacrolimus, have been associated with impaired insulin secretory function and reduced insulin sensitivity, contributing to the development of IR and new-onset diabetes in patients with KT^13^. The pathophysiological mechanisms are still unclear, but strategies such as the use of low-dose tacrolimus or everolimus have been proposed as an alternative to reduce disturbances in carbohydrate metabolism^14^. The association of BMI, abdominal subcutaneous adipose tissue, and visceral adipose tissue with IR is well known^15^, but information on this association in KTRs is lacking, making our results a relevant finding. Several studies have reported the relationship between hypogonadism and IR, mainly in the obese population, but there is a lack of studies on this relationship in KT. In our series, we found a negative relationship of HOMA-IR with E2 and T. The association between hypogonadism and IR is complex. Clegg et al. proposed that gonadal steroids mediate body fat distribution and interact with the integrated message of adiposity transmitted to the brain by leptin and insulin, resulting in differential sensitivity to these signals in men and women. Insulin receptors are distributed in discrete brain areas, including the hypothalamus; these receptors mediate food intake and body weight. Testosterone regulates peripheral and central insulin sensitivity through these receptors^16^. In women, E2 protects proopiomelanocortin neurons from IR by increasing proopiomelanocortin neuronal excitability and insulin receptor coupling with TRPC5 channel activation^17^. At 1 year after KT, this correlation was not evident. We propose that despite the improvement of the HPG axis and its result on insulin homeostasis, several factors related to IR appear gradually during KT follow-up. Our results leave open many lines of future research and development.

The strengths of our study are its prospective nature and the inclusion of young patients with adequate renal function, without overweight, obesity, diabetes, neoplasms, autoimmune disease, graft dysfunction or rejection, which minimizes the bias from these factors on the HPG axis and insulin sensitivity. The limitation of our study may be the follow-up time. We propose the assessment of HPG axis alterations, IR and its related factors before KT and for a prolonged time after KT. Although improvement of the HPG axis is expected after KT, the diagnosis and treatment of persistent hypogonadism and concomitant metabolic disorders (such as IR) should be established to improve diagnosis.

## Conclusion

Successful KT is related to the restoration of gonadal axis function during the first year. Hypogonadism in the early period after KT has a negative correlation with IR; however, despite improvement of the gonadal axis at 12 months, IR persists, and the relationship becomes less significant. Factors such as BMI and tacrolimus dose have a direct association with HOMA-IR at 12 months after KT. Regardless of gonadal steroid levels, several factors could promote IR in long-term TK. The diagnosis and treatment of hypogonadism, IR, and its related factors may improve the prognosis of KTR.

**Abbreviations**
CKD: chronic kidney disease
KT: kidney transplantation
KTR: kidney transplant recipient
HPG: hypothalamic-pituitary-gonadal
LH: luteinizing hormone
FSH: follicle stimulating hormone
E2: estradiol
T: testosterone
BMI: Body Mass Index
HOMA-IR: homeostasis model for insulin resistance
IR: insulin resistance
